# The Italian National Surveillance System for Occupational Injuries: Conceptual Framework and Fatal Outcomes, 2002–2016

**DOI:** 10.3390/ijerph17207631

**Published:** 2020-10-20

**Authors:** Giuseppe Campo, Luca Cegolon, Diego De Merich, Ugo Fedeli, Mauro Pellicci, William C. Heymann, Sofia Pavanello, Armando Guglielmi, Giuseppe Mastrangelo

**Affiliations:** 1 Department of Medicine, Epidemiology, Occupational & Environmental Hygiene, National Institute for the Insurance of Work Related Injuries (INAIL), 00144 Rome, Italy; g.campo@inail.it (G.C.); d.demerich@inail.it (D.D.M.); m.pellicci@inail.it (M.P.); a.guglielmi@inail.it (A.G.); 2 Public Health Department, Local Health Unit N.2 “Marca Trevigiana”, 31100 Treviso, Italy; 3 Institute for Maternal & Child Health, IRCCS “Burlo Garofolo”, 34137 Trieste, Italy; 4 Azienda Zero, Epidemiological Service, 35100 Padua, Veneto Region, Italy; ugo.fedeli@azero.veneto.it; 5 Florida Department of Health, Sarasota County Health Department, Sarasota, FL 34237, USA; William.Heymann@flhealth.gov; 6 Department of Clinical Sciences Florida, College of Medicine, State University, Sarasota, FL 34243, USA; 7 Department of Cardiac, Thoracic, Vascular Sciences & Public Health, Padua University, 35128 Padua, Italy; sofia.pavanello@unipd.it (S.P.); Giuseppe.mastrangelo@unipd.it (G.M.)

**Keywords:** work related injuries, occupational incidents, fatal events, surveillance, workplace, vigilance, agriculture, construction, economic incentives, health and safety at work, occupational regulations

## Abstract

*Background:* A national database of work-related injuries has been established in Italy since 2002, collecting information on the injured person, his/her work tasks, the workplace and the risk factors contributing to incident dynamics, according to a model called Infor.Mo. *Methods:* A descriptive study of occupational fatal injuries, excluding work-related fatal traffic injuries, that occurred in Italy from 2002 to 2016 (15 years) was performed. *Results:* Among 4874 victims involved, all were males, mainly >51 years of age (43.2%), predominantly self-employed (27.8%) or workers with non-standard contracts (25%). About 18.4% and 17.3% of fatal events occurred in micro-enterprises belonging to, respectively, construction and agriculture. A wide range of nationalities (59 countries in addition to Italy) was identified. Overall, 18.9% of work-related fatal injuries were due to some form of hazardous energy—mechanical, thermal, electrical or chemical—that was normally present in the workplace. Workers’ falls from height (33.5%), heavy loads falling on workers from height (16.7%) and vehicles exiting their route and overturning (15.9%) were the events causing the greatest proportion of occupational fatal injuries in the present study (from 2002 to 2016) and in the initial pilot phase, focused on years 2002–2004, with a similar distribution of fatal events between the two time periods. The activity of the injured person made up 43.3% of 9386 risk factors identified in 4874 fatalities. Less common risk factors were related to work equipment (20.2%), work environment (14.9%), third–party activity (9.8%), personal protective equipment/clothing (8.0%) and materials (3.7%). The activity of the injured person remained the most relevant contributing factor even when the incident was caused by two or more risk factors. *Discussion:* Occupational fatal injuries occurred mainly in small size firms (up to nine employees) in hazardous workplaces. Small companies, which account for 68% (2888/4249) of all firms in the present study, generally have fewer resources to remain current with the continuously evolving health and safety at work regulations; moreover, these firms tend to be less compliant with health and safety at work regulations since they are less likely to be inspected by occupational vigilance services. *Perspectives:* An approach being introduced in Italy relies on the use of economic incentives to promote safe and healthy workplaces. The comparison of pre-intervention and post-intervention rates of work-related injuries by means of interrupted time series analyses could detect whether the intervention will have an effect significantly greater than the underlying secular trend.

## 1. Introduction

### 1.1. Background

**Figure 1:**
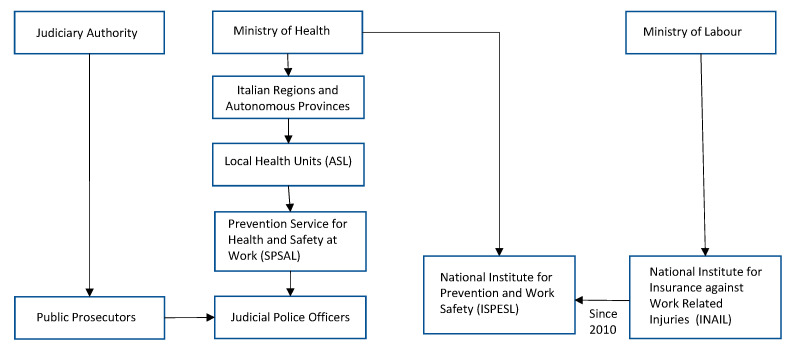
Judicial System, Departments of the Italian Government and Local Occupational Health and Safety Authorities (Hierarchical level: Higher → Lower).

In Italy, occupational incidents are managed by the Judiciary System (breaches of law) and by two governmental departments: The Ministry of Health for prevention of occupational diseases and work-related injuries and the Ministry of Labour for compensation of victims. The hierarchical levels within each body and their relationship with one another are shown in Figure 1. 

The Italian National Health Service aims to ensure healthy and safe working conditions with the involvement and contribution of the employees (law *n*. 833 of 1978). The responsibility for health protection is shared between the Ministry of Health (MoH) and the various Italian Regions and Autonomous Provinces, the former framing the level of support set at national level and supervising an effective delivery of worksite health protection while the latter ensuring health within their catchment area through the respective local health units (ASL, Italian abbreviation) and hospitals. The Health & Safety at Work (HSW) Service (Italian abbreviation—SPSAL—the name varies across the various Italian regions) is a unit of ASL’s public health departments with the task of overseeing the observation of HSW regulations within the catchment area of the respective ASL. 

SPSALs are the main HSW authorities at the local level in Italy. In compliance with the Legislative Decree 758/1994, SPSAL officers are empowered to act as judicial police officers (UPG, Italian abbreviation), operative arms of public prosecutors. Workplace inspections by SPSAL may be scheduled activities—targeting a total of 136,909 Italian companies in 2016 [1]—or actions triggered following notifications of non-compliance with HSW norms by worker representatives (e.g., union representatives), UPGs or public prosecutors. 

In the event of non-fatal occupational injuries requiring less than 40 recovery days off work, the goal of the Italian legislation is primarily ensuring compliance with HSW regulations by supporting business activities. Vigilance interventions start with an official workplace inspection by SPSAL representatives (technicians and/or occupational health doctors) and end up with the issuance of a written report outlining any infringements of HSW norms by the firm. Should the company fail to comply with HSW regulations, SPSAL officers provide technical support, fixing also a deadline to address the workplace violations. A second inspection is then conducted to verify the implementation of the corrections due. If the failure is removed, the infringement is extinguished by payment of an administrative fine. Otherwise, in the case of defaulting at a second inspection, the firm is charged with a criminal offense and faces legal prosecution as well as coercive measures. 

The most severe (with ≥40 recovery days off work) and fatal occupational injuries are presumed to be criminal offenses, unless it is proven that all HSW measures were observed. These events cannot be addressed via administrative penalties. Following prompt notification by Accident & Emergency Hospital Services, SPSAL officers immediately conduct investigations in the worksite to detect the risk factors that may have contributed to or directly caused the event. The prosecutor judge will decide whether a prosecution case should be formally opened in court based on evidence collected during the investigation, and in particular breaches of Laws, Legislative Decrees or Decrees of the President of the Italian Republic and Ministerial Decrees included in the Legislative Decree N. 81 of 2008. The latter decision is influenced by the amount of evidence available; the Italian penal system pursues consistent application of the law, allowing limited discretion by judges as to whether a case should be pursued [2,3]. Prosecutors are empowered to request collection of further evidence of regulation breaches where appropriate [4].

As shown in Figure 1, the national reference institution for HSW was the Italian National Institute for Prevention and Work Safety (ISPESL, Italian abbreviation) introduced by law 833/1978 as a national body affiliated with the Italian National Health Service and now incorporated within the National Institute for Insurance for Work Related Injuries (INAIL, Italian abbreviation). Over the course of the 120 years elapsed since its establishment (law *n*. 80 of 17 March 1898), INAIL has progressively changed its tasks, providing now an integrated system of protection for victims of work related injuries or occupational diseases, including preventive and research actions at the workplace, medical services, financial support, rehabilitation and reintegration to social life and work. However, the INAIL insurance scheme still compensates workers for work related injuries and diseases, including payment of workers’ wages. Furthermore, INAIL is also liable for collecting statistics of work-related injuries and occupational diseases. 

### 1.2. Aims

The Italian national surveillance system for occupational fatal injuries was conceptualized and developed within the above framework. The system has been called Infor.Mo (standing for “Infortuni mortali”, work-related fatalities in Italian). We herewith describe the development, main features and results achieved by Infor.Mo, through a qualitative analysis of occupational fatal events in Italy.

## 2. Methods

### 2.1. Development of the System

According to INAIL reports, non-fatal occupational events diminished during the 1990s while this was not the case for fatal injuries, thus suggesting both a reporting bias—a systematic error of underreporting of minor compared to major injuries [5]—as well as the need of further information on causality of work-related fatalities for preventive purposes. Therefore, in 2000, in cooperation with the 20 Italian Regions and Autonomous Provinces, ISPESL launched a nation-wide research project to monitor fatal and serious workplace incidents. In the same year, INAIL launched a similar project in cooperation with the Social Partners. The latter are joint committees of worker and company management representatives mainly of small and medium size industries. 

On 25 July 2002 INAIL, ISPESL and the Conference of Regions and Autonomous Provinces of Italy signed a consensus agreement aimed at integrating the two above projects into a single Integrated Health Information System for Health Protection at Work, articulated across all 20 Italian Regions. The project was approved and funded by the Italian MoH (MoH Research, Art. 12 e 12 bis D.Lgs. 502/92).

The first complex step of this integrated project was the arrangement of shared working tools. The will of each institution to proceed in partnership with each other and the intense joint activity of their experts produced a unified consensus approach. The agreed model is divided into two sections:

- Section 1 is a general description of the incident, the injured persons and the workplace/work tasks.
- Section 2 is a description of the incident dynamics. This section, initially named “Learning from mistakes” (“Sbagliando s’impara” (SSI, Italian abbreviation), was inspired by Northern European experiences [6,7,8] and developed within the research tasks of ISPESL [9,10].

The model was planned as an information tool to be used during workplace investigations conducted by UPGs to collect evidence on the causes of an incident. 

Since the surveillance system Infor.Mo includes several professional figures (UPGs of HSW and researchers of INAIL) and institutions (Regions, ASLs, INAIL), the development of a database application became necessary. An individual central national database was therefore created, archiving all information on major work-related incidents investigated by Infor.Mo. The electronic database is accessible via Internet from each local station affiliated with the research network. Data are stored anonymously, adopting various strategies to ensure protection of sensitive information according to the Italian privacy law. An open access website was also created to share information about this project: surveillance, technical materials, publications and training opportunities for companies.

An operational manual was published and distributed to operators/users, belonging to SPSAL (HSW officers), ISPESL (coordination) and INAIL (data mangers). Around 1000 operators involved in the project were trained in order to achieve or improve their professional competence, maintaining the highest standard of homogeneity of skills across different institutions (Regions, ASLs and INAIL). 

The INAIL researchers and the operators belonging to Infor.Mo were classified as follows:

- Type A: HSW Officers, assigned to incident investigation
- Type B: Operators with coordination tasks at the regional level
- Type C: Data managers (INAIL researchers)

Moreover, within companies adopting Infor.Mo, the professional figures appointed to use the model are mainly the Responsible for Prevention and Protection Services. Training courses were held in more than 220 companies belonging to the following production sectors: manufacture of metal products, construction, pharmaceutical chemistry, wood industry, transport and storage, steel and foundries, electricity production and waste management activities. Over 90% of these were small and medium (up to 249 employees) firms. In particular, 56% were micro-enterprises (maximum nine employees), 35% mid-size companies (10–249 employees) and 9% big companies (≥250 employees). 

Each type of operator received different training, following identification of their formative needs, teaching methods, teaching staff, learning assessment tools and evaluation system. For SPISAL officers, the training was included in the annual credits for mandatory continuous medical education. For small- and medium-sized industries, the model was supported by trade unions. 

Lastly, Regional Coordination teams (warranting the completeness and quality of information collected at regional level) and a National Coordination team (including representatives of MoH and INAIL) were established. Within the latter, each methodological and organizational aspect of the surveillance system was discussed and agreed upon. 

The initial pilot phase, related to calendar years 2002–2004, was broken down into two parts.

- January 2002–October 2003: Retrospective data collection, from investigations already conducted by SPSAL and local officers of INAIL
- November 2003–December 2004: Prospective data collection, following the above-described surveillance model, with higher standards of data collection, completion and quality.

Data collection has continued up to today.

A second objective of Infor.Mo is to support companies in workplace risk assessment, whenever a fatal event occurs. Furthermore, Infor.Mo has been extended to cover a set of serious non-fatal incidents, with the aim of expanding the detection of risk factors in sectors with frequent, although non-fatal, events (for example, in the manufacturing industry or wood industry). Nonetheless, the Infor.Mo model could be applied to any type of incident, including near misses. The corporate health protection services can use the above model to assess each incident within their company, highlighting the respective risk factors (to be removed with appropriate interventions) and reviewing the risk assessment. 

### 2.2. Quality Checks

The critical points and difficulties encountered by operators/users were mostly related to the application of the Infor.Mo model. Therefore, further specific professional training was provided in order to support the Regional Scientific Managers and Local Referents of INAIL in carrying out content checks. Moreover, a reference guide was created to define criteria and operational indications to perform the content checks, based on what emerged from this phase of professional update. To further improve the quality of the database, supervision at the national level was set up for events already confirmed at the regional level. Several meetings were held between operators of all regions to analyze the events and adopt appropriate interpretative solutions. The expected competencies of SPSAL officers are updated by annual training. To date, more than 60 courses (10 of which were e-learning) have been run, at both national and local level.

### 2.3. Conceptual Framework 

The structural components of an injury (all occurring in a very short time interval) are:

- the incident;
- the contact; and
- the damage.

The reconstruction of the incident dynamics follows the classic backward path used in the judicial investigative process. Starting from the last event in chronological order (the damage), the investigation proceeds to its root causes. Once identified, the three components of the injury (event, contact and damage) will be described by the analyst, who will also categorize them as follows.

The incidents causing work injuries can be classified in two categories.

- Incidents due to inadvertent and sudden release of hazardous energy (e.g., any electrical, mechanical, hydraulic, pneumatic, chemical, nuclear, thermal, gravitational or other energy that provides power to a system to perform work) following an unintentional action altering the system (procedures, techniques, designs, methods and equipment) of hazardous energy control.
- Incidents due to hazardous energy that was normally present in the workplace. Examples are mechanical energy (moving gears and machine appliances not adequately isolated), thermal energy (open flames), electrical energy (electrical wires without insulation or with defective insulation) and chemical energy (open containers of strongly irritating or caustic substances). When the hazardous energy is normally present in the workplace and is directly accessible, its contact with the worker coincides with the event. These incidents usually occur in poorly maintained workplaces.

For both categories of incidents, the model Infor.Mo labels the “mode of occurrence” (for example, fall of workers from height, fall of heavy loads onto workers, vehicles deviating from their route and overturning, etc.) according to a list adopted by the European Statistic of Incidents at Work (ESAW), a system born in the 1990s to record work-related injuries data in Europe. Only for accidents due to the inadvertent release of hazardous energy, Infor.Mo adds the “material agent of an event”, information which varies according to the reported mode of occurrence (e.g., for “fall of worker from height”, “fall of heavy loads on workers” or “vehicles deviating from their route and overturning”, the material agent describes “from where he fell”, “from where the heavy body loads” or “the vehicle which lost control”). In summary, the combined information of “mode of occurrence” and “material agent of an event” provides specific information on incident dynamics.

The conceptual framework we used to classify factors contributing to a work-related injury is a classification scheme allowing a systematic injury description according to multiple risk factors contributing to the injuries. This resulted in a matrix consisting of six major categories of contributing risk factors.

1. Activity of the injured person: Inappropriate actions of the injured person during the incident were: errors in procedure/operation, incorrect use of equipment and improper use of equipment.
2. Third–party activities: Inappropriate actions performed by third parties (other workers or other people present on the incident scene) were the same as reported in Point 1.
3. Machines, tools and plant: Equipment of any type (or part of it) which in the incident dynamics presented critical issues such as set-up (lack of equipment security, insufficient safety of equipment, presence of dangerous elements, removal of protections, tampering with protections, etc.) or operating issues.
4. Materials: Storage, structural characteristics and transformations were the critical items found in the material being processed during the incident dynamics.
5. Environment: Critical issues found where the events developed were absence of barriers, protections, parapets, armor and inadequate signage; absence of safe routes; presence of bulky dangerous elements; presence of electrical wires and electrical line; absence of suitable lighting; presence of gas and vapors; presence of liquids (water, oils, etc.); and other problems of the workplace.
6. Personal protective equipment (PPE) and clothing: Critical issues during the incident dynamics were non-used yet available PPE; PPE not provided; structural inadequacy of PPE; incorrect use of PPE; and wear and tear.

Each of the six category of contributing risk factors can behave as either “determinant” or “modulator” (see below), and each determinant or modulator can in turn be specified as “state” or “process” (see below).

- Determinants and Modulators: Every factor acts as a determinant if it increases the risk of incidents. The factor serves as a modulator if it is able to increase the resulting biological damage.
- State and Process: Each factor is defined as “state” if it is present at the beginning of the incident dynamics and remains unchanged during the dynamics. Every factor occurring during the incident dynamic is defined as “process”.

Safety issue: This variable explains the reasons a particular category of risk factors was identified by the analyst as an item influencing the incident dynamics under examination. Each of the six categories has its own safety problems, regardless of whether the corresponding risk factor was either determinant or modulator, state or process.

## 3. Results

In the three sections below, we report detailed information on:

- the injured person and workplace/job (Table 1);
- the work-related injury (Table 2 and Table 3); and
- the risk factors involved, according to the Infor.Mo model (Table 4, Table 5, Table 6, Table 7, Table 8 and Table 9).

Only fatal events passing quality check were selected for the analysis. The quality checks verified the consistency between the information drawn during the investigation and definitions provided by Infor.Mo analysis model. 

In an observation period of 15 years (2002–2016), the cases of fatal work-related incidents were 4874, an average of 325 cases/year. The number of risk factors detected during workplace investigations were 9386, an average of 1.9 factors/event. Descriptive data analysis was performed.

The number of cases present in the archive Infor.Mo does not cover the total number of fatal work-related injuries that occurred in Italy. For example, road incidents were excluded because causality investigation was not conducted by SPSAL. On the other hand, some events in the database involved people not formally classified as workers (for example, pensioners or family members) for whom an investigation was conducted because an occupational risk was involved in their injuries.

### 3.1. Analysis of Injured Persons 

Occupational fatal injuries are predominantly an issue of male workers. Accordingly, INAIL statistics reported that the victims of these incidents are males in 98% cases [11]. 

Table 1 shows the main variables traditionally considered in the injury statistics (characteristics of the injured worker, occupation, work task and company sector). The corresponding percentages among the entire Italian workforce, recorded by the Italian Office of National Statistics (ISTAT) in 2016 [12], are reported in the last column of Table 1. 

The most represented age class was 41–50 years among both injured persons (24%) and Italian workforce (30%). It is worth mentioning that, in the most advanced age group (≥65 years), the percentage of occupational fatal injuries was 14%, while that of the Italian workers was only 2%. 

Seniority is defined as the length of time an individual has served in a job-task, regardless the number of years of work spent in the company. According to our data, 70% of occupational fatal injuries occurred among experienced workers (seniority >3 years). As can be seen, more than 5% of fatal events happened within the first seven days of work in a company and an additional 2.5% occurred within a month, suggesting either a lack of experience of the worker or regularization of his employment position after the incident has occurred. Such percentages were not available for the Italian employees.

Wide variation of nationality was found among foreign workers: 59 different nationalities were identified, predominantly Romanians (4.3%), Albanians (2.8%) and Moroccans (1.6%). Fatal work-related injuries were much more prevalent among Italians (85%) in the present study. According to ISTAT, the percentage of workers of Italian nationality was 90% in the country during 2016. Regarding citizenship, the item “Romania” was not found because the official source aggregates the states of the European Union. The only available information regarded workers from Albania and Morocco with, respectively, 0.8% and 0.6% employees in 2016 [13].

Employees with permanent contract (47%) and self-employed/business owners without employees (14%) were the main working statuses associated with occupational fatal events. At the time of the investigation, 7% injured persons did not have a regular work contract. The proportion of pensioners among occupational fatal incidents is explained by the fact that ASL are obliged to conduct investigations regardless of whether the person is covered by regular occupational insurance including also work-related injuries. With regard to employment relationship, our data use an ad hoc classification (also including pensioner, precarious workers, non-standard (irregular) employees and contributing family workers) rather than the classic forms of employment. Furthermore, in Italy, there were numerous changes in the labor market, with consequent variations in the types of contracts during the period under investigation. Since they were highly aggregated, no comparison data are reported. 

Likewise, job tasks are categorized according to internal classifications rather than the International Standard Classification of Occupations (edited by the International Labour Organization) or the classification of economic activities ATECO (edited by the Italian Office for National Statistics) that is the Italian national version of the European nomenclature, NACE Revision 2 based on European Regulation (EC) no. 1893/2006 [14]. Therefore, no data are reported for comparison in the last column of Table 1. 

Sixty-eight percent of fatal work-related incidents occurred in small companies (<10 employees), normally featured by lower standards of workplace heath protection. According to ISTAT, the percentage of small firms in Italy was 45% in 2016. By contrast, in firms with 250 and more employees, the percentage of fatal events was 2.5% compared to 22.1% in ISTAT data. 

Agriculture and construction were confirmed to be the economic sectors where occupational incidents produced the greatest damage, with higher rates of fatal events, balanced between skilled and general work tasks. By contrast, the transport industry was under-represented in this database. 

### 3.2. Analysis of Work-Related Events

Most occupational fatal injuries were caused by incidents due to inadvertent and sudden release of hazardous energy not adequately controlled (N = 3953, 81.1% of the total). Incidents due to hazardous energy that was normally present in the workplace occurred in less than a fifth (18.9% = 921/4874) of total events. 

Table 2 shows the distribution of occupational fatal events according to mode of occurrence. About half of the events were caused by workers’ falls from height (33.5%) or heavy loads falling on workers from height (16.7%). In the third position were events due to vehicles deviating from their lane and overturning (15.9%). These first three incident modes accounted for about 2/3 of the entire database.

For the first three most frequent modes of occurrence, Table 3 shows the corresponding material agents of the incident. Almost a third of falls from height (29.5%) occurred from roofs and 28.8% from work equipment at height (primarily scaffoldings and portable stairs). Falls of heavy loads on workers from height occurred mainly from lifting/transporting machines (17.4%) and from storage areas where the materials were not safely stacked (15.6%). Vehicles losing control were mainly agricultural and forestry machinery (48.9%), lifting/transporting machines (18.3%) and earth moving as well as road construction machines (13.1%).

### 3.3. Analysis of Contributing Factors

Contributing factors identified in the incident dynamics are analyzed in detail in this section. Overall, 9386 risk factors were identified for the total 4874 occupational fatal cases investigated.

Table 4 shows the frequency of risk factors according to the six categories proposed by the Infor.Mo model. The activity of the injured person constituted the most frequently recognized risk factor of occupational fatal events (43.3%). Work equipment (20.2%) and environmental factors (14.9%) represented the following two most frequent hazardous conditions, whereas the roles of third-party activity (9.8%), PPE/clothing (8.0%) and materials (3.7%) were more marginal.

Table 5 shows the six categories of contributing factors classified as “determinant” or “modulator”, as well as “state” or “process”, according to the role played in the incident dynamics. For these mutually exclusive characteristics, row percentages (rather than column percentages of the above tables) were calculated. Except for PPE/clothing, which mainly played as modulator (82.5%), all other categories of factors acted largely as “determinants”. The activities of the injured person and third parties are by definition measures of process. The other four categories of contributing factors were more frequently classified as “state” rather than “process”. The high frequency of states (especially “tools, machines and equipment”, “materials” and “PPE and clothing”) is of great importance for prevention purposes since the states are by definition identifiable before the event occurs and therefore are completely independent from it. Nevertheless, albeit not always possible and often demanding, processes can still be improved, as endorsed by programs for the promotion of quality and the adoption of best practices.

Table 6 shows the distribution of the safety issues identified for each of the six categories of contributing factors. The safety issues associated with the “Activity of injured persons” included procedure or operative errors (75.7%) and incorrect (18.5%) or improper (5.8%) use of equipment. Records which did not present any safety issue (*N* = 44) were excluded. Each type of safety issue could be broken down into detailed operational aspects. For example, 38.8% of procedural errors were due to incorrect practices usually adopted within the company and 30.9% to the lack of HSW courses in the workplaces. Unfortunately, this evidence is weakened by a percentage of missing values as high as 43% ((1741‒3046)/3046; see figures in bold, Table 6). Similar considerations apply to the safety issues related to “Third-party activities”, where the percentages of missing observations for procedural errors and incorrect or improper use of equipment were 43%, 49% and 56%, respectively (14 records with missing information on safety issues were excluded). Nonetheless, these results call for improved training of workers.

As for “Tools, machines and plants”, the set-up problems are much more frequent (85.6%) than the operating problems (14.4%). In detail, 72% set-up problems are due to lack, inadequacy or removal or tampering of protections necessary to work under safe conditions. Interestingly, out of 1900 observations, only 26 were missing values. These results are in line with the preponderance of the states over processes, as reported in Table 5, and confirmed the high predictability and preventability of the incidents examined. The contributing factor “Materials” presented three safety issues (one missing), related to their storage (47.2%), their structural characteristics (42.9%) or their transformations (9.9%). The safety issues related to the “Environment” were mainly the absence of barriers, protections, parapets and armor (36.9%), along with inadequate signals and absence of safe routes (21%); the latter issue affects the movements of pedestrians and vehicles. Three missing values were found among 1400 observations on workplace conditions. Concerning “PPE and clothing”, there are two safety issues (29 missing): non-use but available PPE (57.9%) and PPE not provided (32.3%).

Table 7 shows a two-way classification of work-related fatalities, stratifying the six categories of risk factors by their number causing the incident. The last column of Table 7 displays the distribution of event by number of risk factors (column percentage). When only one factor was identified, the corresponding category of contributing factors was the activity of the injured person (in 58.4% fatal events). The activity of the injured person was the most relevant contributing risk factor even when the event was caused by two or more risk factors. Overall, 4067 out of 9386 risk factors (43.3%) concerned the activity of the injured person.

The 9386 risk factors detected in 4874 work related incidents equaled 1.9 factors/event. Therefore, from the collection of 9386 contributing factors, we selected all combination of two factors. The total number of two-factor combinations, *f*(2), is equal to the sum of the binomial coefficients

(1) $$f\left(2\right)=\overset{7}{\underset{n=2}{\sum }}\frac{n!}{k!\left(n-k\right)!}$$

where *k* = 2 (pairs of factors) and *n* = 2–7 (number of risk factors by fatal event; there were seven fatalities with seven risk factors). 

Table 8 reports the frequencies of combinations of two risk factors of occupational fatal events. The association of “Activity of the injured person” and “Tools, Machines, Plants” was the most common, collecting 22.4% of all 6085 associations. “Activity of the injured person” and “Environment” came next, making up 14.5% fatalities. It is worth noting that, according to the Infor.Mo model, each fatality could have two or more risk factors of the same category (e.g., two factors both classified as “Activity of the injured person”), which may represent different entities to be treated separately from a preventative perspective.

Based on the data in Table 8, Table 9 displays the combinations of each risk factor with another risk factor in decreasing order of frequency. As can be seen, the activity of the injured person was of utmost importance for occupational fatal events because it was always present in each combination of two risk factors. 

## 4. Discussion

### 4.1. Key Results

There was no model in Italy that could guarantee the uniformity of analysis for occupational fatal events. A single model was difficult to define because of the need to overcome different established practices and experiences of the operators/inspectors. The latter is one of the most interesting aspects of the present project. Nonetheless, Infor.Mo has been generally believed or recognized to be valid or correct compared to previous methods. Therefore, a national database of occupational injuries was established in Italy in 2002, collecting information on injured persons and workplace/work tasks as well as risk factors contributing to incident dynamics, according to the model Infor.Mo. The model is currently in use by SPSAL officers in their investigations triggered by the prosecutor judge. Having passed accurate quality controls, the present national database of 4874 occupational fatal injuries constitutes a solid base for their description and interpretation of fatal work-related injuries which occurred in Italy during 2002–2016 (15 years), except for traffic work-related injuries.

Victims involved were all male workers, mainly >51 years of age (43.2%), predominantly self-employed (27.8% being micro-entrepreneurs without/with employees or membership in a worker-cooperative) or non-standard workers (25% including pensioners, contributing family workers and undocumented or precarious/fixed term workers). About 18.4% and 17.3% occupational fatal fatalities occurred in micro-enterprises belonging to, respectively, construction and agriculture, sectors already known for the high risk of injuries. A wide range of nationalities (59 countries excluding Italy) was identified among injured workers. 

Less than a fifth (18.9%) of fatalities were due to some form of hazardous energy—mechanical, thermal, electrical or chemical—normally present in the workplace; the relevant most common causes were contact with moving gears (5.5%) and direct electrical contact (4.1%). Since a hazardous energy, capable of causing serious damage or death, was directly detectable and manageable during the risk assessment, the latter incidents were easily predictable and preventable. On the other hand, about 40% events were due to indicators of “state”, yet present at the beginning and remaining unchanged during the incident dynamics. Tools, machines, plants and in addition environment acted mainly as “state” risk factors. The assumption that occupational incidents are the result of chance would frustrate any attempt to prevent them. Rather, occupational injuries are preventable because they are events generally determined by pre-existing worksite conditions [9].

Workers’ falls from height (33.5%), heavy loads falling on workers from height (16.7%) and vehicles deviating from their route and overturning (15.9%) accounted for about 2/3 of the entire database. Agricultural and forestry machinery made up about half the vehicles losing control; others were lifting/transporting machines, earth moving and road construction machines. 

The activity of the injured person accounted for 43.3% of 9386 risk factors detected in 4874 fatal injury cases. Less common risk factors were related to work equipment (20.2%), work environment (14.9%), third–party activity (9.8%), PPE/clothing (8.0%) and materials (3.7%). The activity of the injured person remained the most relevant contributing factor even when the events were caused by two or more risk factors. The combinations of two distinct risk factors always involved the activity of the injured person. Concerning the safety issues associated with the activity of injured person, errors in procedure/operation and incorrect or improper use of equipment ranked first (75.7%), second (18.5%) and third (5.8%), respectively. 

### 4.2. Interpretations of Findings

Treating workplace fatalities as potential criminal offenses is unusual in several countries. In Italy, this approach is driven by the default presumption that a fatal occupational event is the aftermath of a crime and therefore the case needs to be assigned to an independent adjudicator, such as the Public Prosecutor, who will have to decide whether to formally open a court trial. However, prosecutors are guided by specific official procedures which lead their decisions, ensuring a consistent application of the law [2]. Unfortunately, the full number of criminal cases opened in court by public prosecutors for breaches of HSW regulations was not possible to be retrieved, since there are no publicly available national databases storing such information and, moreover, the restrictions of the Italian privacy law prevented us from reconstructing this figure from our database. Nonetheless, we still managed to retrieve 273 criminal sentences (out of 350 court cases) issued between 1999 and 2015 by the Italian Supreme Court, involving the constructions sector [15]. Injuries (49%), death (41%) and breaches of HSW regulations (11%) were the main outcomes investigated by public prosecutors. Workers’ falls from height (43%) and heavy loads falling on workers from height (13%) were the most common modes of injury occurrence. Workers comprised 80% of the victims. Defendants were mainly employers (51%), managers (12%) and those responsible for prevention and protection services (9%). The outcome of the respective sentences ranged from penalties for employers (46%) or other defendants (43%) to acquittal for the employers (5%) or other subjects (7%). The overall percentage of falls from height (43%) is consistent with the constructions sector; such percentage was 38% in the period 1999–2010 and increased to 48% from 2011 to 2015 [15].

In the present investigation, occupational fatal injuries occurred mainly in small firms with hazardous workplaces and use of irregular workers, suggesting that some employers may seek out offsetting reductions in their labor costs. In agreement with the present findings, higher rates of occupational fatal/severe events in smaller rather than larger worksites were observed among the 17,481 occupational fatalities investigated in the US by the Occupational Safety and Health Administration during 1992–2001 [16]. Although this seems to suggest that smaller workplaces generate higher occupational risks, several additional explanatory factors may be involved [17]. First, since they cannot benefit from large-scale economies, small companies have fewer resources to remain current with the continuously evolving HSW norms [17,18]. Second, the burden of HSW regulations is heavier on small business activities than large ones [17]. Third, smaller worksites are frequently difficult to reach and less likely to be inspected by occupational vigilance services [17]. 

To assess the effects of occupational safety and health regulation and legislation enforcement activities, such as inspections for preventing work related injuries and occupational diseases, a review included 23 studies: two randomized controlled trials on 1414 workplaces, two controlled before–after studies on 9903 worksites, one interrupted time series with 6 outcome measurements, 12 panel studies and 6 qualitative studies with 310 participants. Although with low level of evidence, workplace inspections were seemingly able to reduce incidents more in the long run than in the short run. The effect size was stronger with focused rather than general inspections. The effect of fines and penalties remained undefined. Since the quality of the evidence was low to very low, these conclusions were tentative and could be easily amended in the future by improved research [19]. 

There was an urgent need for better designed evaluations to establish the effects of existing and novel enforcement methods. A more recent systematic review of the literature covering calendar years 1966‒2017, including 45 journal articles and 16 reports of the grey literature, was undertaken to assess whether HSW legislative and regulatory policies could reduce industrial injuries and fatalities. The review indicates that legislative and regulatory policies may reduce injuries and fatalities and improve compliance with HSW regulation. The evidence for improvement was moderately strong [20]. 

Nevertheless, regarding intervention on small enterprises, a review of studies published before 2006 reported a lack of evaluation, in terms of both effect and practical applicability. The most effective preventive approaches seemed to be simple and low-cost solutions, disseminated through personal contact [21]. A later systematic review was undertaken to identify effective interventions to improve HSW conditions in small businesses. Five studies of medium or high quality were identified. A combination of training and safety audits, as well as a combination of engineering, training, safety audits and a motivational component, showed limited evidence of improving safety outcomes. However, stronger levels of evidence are required to draw appropriate conclusions and recommendations [22].

Workers’ falls from height, heavy loads falling on workers from height and vehicles exiting their route and overturning were the events causing the greatest proportion of occupational fatal injuries in either the present study (2002–2016) and in the initial pilot phase, related to years 2002–2004 [23]. The distribution of these work-related incidents was similar between the two periods, being 33.5% versus 31.9%, 16.7% versus 15.1% and 15.9% versus 12.7%, respectively, for fall of workers from height, loads falling on workers and vehicles losing control. Most of the above events occurred in small firms belonging to Constructions and Agriculture. 

Despite being a critical business for all countries in the world, construction projects cause high injury risk. According to van der Molen et al. [24], the vast majority of technical and human factors and organizational interventions recommended by standard safety textbooks, consultants and safety courses, have not been adequately evaluated. To assess the effectiveness of interventions for preventing work related injuries among construction workers, a systematic review was conducted using published findings from the earliest available dates through June 2006. Findings from a safety-campaign study and a drug-free-workplace study indicated that both interventions significantly reduced the level and the trend of incidents. Three studies evaluating legislation did not find decreased levels of non-fatal or fatal injuries in the construction industry [25]. In a more recent systematic review on 17 interventional studies (14 interrupted time series and 3 before-after studies) collected up to 1 April 2017, a significant effect was measured in the year immediately after the intervention and a sustained effect was assessed as change in time trend before and after the intervention. The association between regulatory measures at national and local level and immediate as well as sustained effect on fatal and non-fatal occupational events was also not consistent and safety training did not translate into a significant reduction of non-fatal injuries. Economic incentives to companies may reduce the rates of non-fatal injuries from falls, and multifaced interventions may significantly decrease the initial and sustained incident rate at company level, albeit not at regional scale. Whilst a multifaced drug-free workplace intervention at company level might have decreased non-fatal injuries in the year following the implementation and to smaller extent also in the subsequent years, the implementation of occupational health services did not reduce fatal and non-fatal events [26].

Besides the construction industry, agriculture was the other main area of concern for occupational fatalities in the present study. Accordingly, among 335,000 yearly fatal occupational injuries worldwide, 170,000 were agricultural workers [27]. In a systematic review on interventional studies in agriculture up to June 2006, three randomized controlled trials on 4670 adult participants and two RCT on 6895 children participants did not show any significant reduction of the rates of occupational incidents. While educational interventions may not significantly decrease agriculture injuries, monetary incentives may be effective in reducing them [28]. Regulations enforcing the introduction of protective devices on all tractors in Sweden were not beneficial in reducing fatal injuries, while the same measures for new tractor machines did translate into fewer fatal events [29]. 

## 5. Conclusions

### 5.1. Limitations

The risk of occupational injuries increases with work-related factors along with individual factors such as age, sex and smoking [30,31,32], physical inactivity and obesity [33,34] and pre-existing conditions [31,33,35,36] including diabetes [37]. Human factors also include workers’ action/behavior related to safety, skills, communication and available supervision, inter-personal and hierarchical relationships, corporate context, unions, internal and external work competition, etc. [14]. The role of personal factors could not be confirmed in the present study because, besides age and sex, the database does not contain information on a worker’s individual characteristics. However, the confounding role of these factors was likely consistent throughout the study period as shown by one observation—falls from height, heavy loads falling on workers and vehicles losing control—occurring with similar percentages between the two time windows. 

The ratio between events recorded in a population sample (numerator) and subjects in the same sample (denominator) with simultaneous assessment of time at risk permits a comparison of work-related incidents among quantitatively different samples of population. However, the lack of denominators allows only a simple statistical analysis (percentages). These findings therefore need further confirmation in studies with precise denominator information.

### 5.2. Perspectives

Small companies cannot benefit from large-scale economies and have therefore fewer resources to catch up with the continuously evolving HSW regulations. Since 2006, the Italian governments have passed legislations supporting INAIL funds designed to incentivize the improvement of HSW standards among small, medium and micro-enterprises [3]. The main economic tools were discounts on the amount firms pay for insurance for work-related injuries and occupational diseases (OT 23 model) [38] and, on the other hand, non-refundable economic incentives (INAIL business support incentives, ISI model) [39]. 

Criteria used by INAIL in the last years to provide OT 23 benefits were the following: adoption or maintenance of appropriately certified HSW management systems; declarations issued by “*Organismi paritetici*” (bilateral commissions of both employers’ and workers’ representatives for cooperation in the field of HSW); procedures for recording near-misses at work; adoption of training activities; social facilities granted to workers; and activation of campaigns in cooperation with ASL against smoking, alcohol and drugs and encouraging a healthy diet. In 2020, new issues were introduced such as reintegrating persons with disabilities into the work force and transporting workers home after night shifts. 

Through annual calls for application, the ISI model aims to support companies in carrying out projects to improve HSW standards, funding companies up to a maximum of 65% of costs. The following projects may be eligible for funding:

- investment;
- adoption of organizational and social responsibility models;
- remediation from asbestos containing materials; and
- other projects for micro and small enterprises operating in specific sectors.

Compensation is paid to companies positively positioned in the ranking, after completion of the project and following a technical-administrative check. The financial resources granted under the ISI model averaged a yearly €232.6 million (± €88.6 million) from 2010 to 2017 [3].

The economic incentives (OT 23 and ISI) are issued with the aim of introducing additional interventions to improve HSW; therefore, companies must demonstrate compliance with the minimum HSW legal requirements to access these monetary funds. Small firms, often demonstrating poor compliance with HSW regulations [40], will receive an economic benefit by following the rules.

It is still unclear whether financial incentives are more successful than HSW regulations to ensure the next sentence healthy worksites and new evidence is needed [41]. A retrospective national study could be conducted collecting INAIL data on injuries and plants at multiple instances over time in order to have at least eight quarters of the year of observations before and eight after the intervention. The comparison of pre-intervention and post-intervention rates of work-related injuries by means of interrupted time series analyses could detect whether the intervention will have an effect significantly greater than the underlying secular trend. 

## Figures and Tables

**Table 1:** Number (N) and column percentage (%) of fatal injuries in relation to the most important characteristics of the injured worker and their company/occupation.

Factors	Classes	N	%	Distribution of Italian Workforce (%) *
Age (years)	15–24	281	5.8	4.3
	25–34	712	14.8	17.9
	35–44	1057	22.0	28.1
	45–54	1176	24.4	30.3
	55–64	926	19.2	17.1
	65+	661	13.7	2.3
	Total	4813	100.0	100.0
Job Seniority	Up to 7 days	206	5.1	NA **
	8 days up to 1 month	100	2.5	
	>1 month up to 6 months	258	6.4	
	>6 months up to 1 year	199	4.9	
	>1 year up to 3 years	459	11.4	
	>3 years	2807	69.7	
	Total	4029	100.0	
Citizenship	Italy	4091	84.6	90.1
	Romania	208	4.3	NA **
	Albania	134	2.8	0.8
	Morocco	77	1.6	0.6
	India	26	0.5	0.3
	Other countries	301	6.3	8.2
	Total	4837	100.0	100.0
Employment Relationship	Permanent (regular) employee	2230	47.0	NA **
	Self-employed without employees	677	14.3	
	Worker in a worker cooperative enterprise	358	7.5	
	Pensioner	341	7.2	
	Irregular and/or precarious worker	339	7.1	
	Self-employed/Owner with employees	285	6.0	
	Non-standard (irregular) employee	206	4.3	
	Fixed-term worker	163	3.4	
	Contributing family worker	143	3.0	
	Total	4742	100.0	
Job	Farmers and Farm Workers	614	14.3	NA **
	Bricklayers, Stonemasons	501	11.6	
	Construction Finishing and Exterior Walls Painting	292	6.8	
	Heavy Goods Vehicle and Truck Drivers	251	5.8	
	Motor Vehicle Mechanics and Repairers	233	5.4	
	Construction of Scaffolding and Tunnel Workers	214	5.0	
	Electrical Equipment Installers and Repairers	170	4.0	
	Unskilled Construction Workers	168	3.9	
	Carpenters and Joiners in Construction	157	3.6	
	Casters, Welders, Carpentry Fitters	149	3.5	
	Unskilled Farm Workers	131	3.0	
	Other 50 Jobs	1422	33.1	
	Total	4302	100.0	
Company Size	Up to 9 employees	2888	68.0	45.3
	10–15 employees	410	9.6	19.7
	16–49 employees	481	11.3	
	50–249 employees	365	8.6	12.9
	250 and more employees	105	2.5	22.1
	Total	4249	100.0	100.0 ^#^
Sectors	Buildings and Civil Engineering Works	823	17.5	2.4
	Crop Farming	729	15.5	NA **
	Building Completion and Finishing	279	5.9	2.3
	Building Installation Services	237	5.0	2.6
	Land, Sea and Air Transport	209	4.4	6.6
	Manufacture of Metal Products	207	4.4	3.1
	Roofing Installation and Roof Construction	153	3.3	0.3
	Agriculture-Related Activities	148	3.1	NA **
	Construction Site Preparation	132	2.8	0.2
	Forestry and Forest Use	127	2.7	NA **
	Other 41 sectors	1661	35.3	82.5
	Total	4705	100.0	NA **

* ISTAT online database 2016. ** NA, ISTAT data not available in this detail. ^#^ Data of small size companies of agriculture sector not included (not available).

**Table 2:** Distribution of fatal accidents by mode of occurrence. Number (N); column percentage (%).

Mode of Accident Occurrence (Categories)	*N*	%
Fall of Workers from Height or into Holes	1635	33.5
Fall of Heavy Bodies on Workers	814	16.7
Vehicles exiting their route and overturning	777	15.9
Contact with Moving Vehicles	360	7.4
Unexpected/Improper Starting of Vehicle, Machine, Equipment, etc.	297	6.1
Contact with Moving Gears	268	5.5
Direct electrical contact	200	4.1
Ejection/emission of solid particles	155	3.2
Development of flames	92	1.9
Other 11 modalities	276	5.7
Total	4874	100.0

**Table 3:** Main material agents detected in most frequent modes of occurrence of fatal accidents at work. Number (N); column percentage (%).

Mode of Occurrence	Material Agent	*N*	%
Fall of Workers from Height or into Holes	Roofs	483	29.5
	Equipment for Working at Height	471	28.8
	Other Parts at Height (Balconies, Openings, etc.)	269	16.5
	Lifting and Transport Machines	64	3.9
	Other Material Agents	348	21.3
	Total	1635	100.0
Fall of Heavy Bodies on Workers	Lifting and Transport Machines	142	17.4
	Materials (in Storage Areas)	127	15.6
	Walls	80	9.8
	Holes, Excavations, Escarpments	79	9.7
	Other Material Agents	386	47.5
	Total	814	100.0
Vehicles exiting their route and overturning	Agricultural and Forestry Machinery	380	48.9
	Lifting and Transport Machines	142	18.3
	Earth Moving and Road Works Machines	102	13.1
	Land vehicles	92	12.5
	Other Material Agents	61	7.2
	Total	777	100.0

**Table 4:** Risk factors for fatal occupational injuries. Number (*N*); column percentage (%).

Risk Factors for Occupational Accidents	*N*	%
Activity of the Injured Person	4067	43.3
Tools, Machines, Plants	1900	20.2
Environment	1400	14.9
Third–Party Activity	919	9.8
PPE and Clothing	754	8.0
Materials	346	3.7
Total	9386	100.0

**Table 5:** Distribution of contributing factors for fatal work accidents according to their role played in the accident dynamics. Number (*N*); row percentage (%).

Factors		Determinant (a)	Modulator (b)	Process (c)	State (d)
Activity of the Injured Person	*N*	3380	687	4067	0
	% ^@^	83.1	16.9	100.0	0.0
Third–Party Activities	*N*	878	41	919	0
	% ^@^	95.5	4.5	100.0	0.0
Tools, Machines, Plants	*N*	1471	429	274	1626
	% ^@^	77.4	22.6	14.4	85.6
Materials	*N*	332	14	110	236
	% ^@^	96.0	4.0	31.8	68.2
Environment	*N*	1269	131	176	1224
	% ^@^	90.6	9.4	12.6	87.4
PPE and Clothing	*N*	132	622	117	637
	% ^@^	17.5	82.5	15.5	84.5
Total	*N*	7462	1924	5663	3723
	% ^@^	79.5	20.5	60.3	39.7

^@^ percentage Column (a) + percentage Column (b) = 100%; percentage Column (c) + percentage Column (d) = 100%.

**Table 6:** Factors contributing to fatal work accidents by category and safety issues. Number (N); column percentage (%).

Factors	Category		Safety Issue	*N*	(%)
Activity of the injured person (*N* = 4067)	Errors in procedure/operation		Usual practice	676	38.8
			Lack of OHS training in workplaces	538	30.9
			Extemporaneous action	455	26.0
			Health status	72	4.1
			**Effective total**	**1741**	**100.0**
			**Total (including missing values)**	**3046**	
	Incorrect use of equipment		Extemporaneous action	180	41.0
			Lack of OHS training in workplaces	136	31.0
			Usual practice	112	25.5
			Health status	11	2.5
			**Effective total**	**439**	**100.0**
			**Total (including missing values)**	**743**	
	Improper use of equipment		Usual practice	67	50.4
			Lack of OHS training in workplaces	39	29.3
			Extemporaneous action	26	19.5
			Health status	1	0.8
			**Effective total**	**133**	**100.0**
			**Total (including missing values)**	**234**	
		**Effective total**		**4023**	
		**Total (including missing values)**		**4067**	
Third-party activity (*N* = 919)	Errors in procedure/operation		Usual practice	103	33.6
			Lack of OHS training in workplaces	77	25.1
			Extemporaneous action	71	23.1
			Communication	55	17.9
			Health status	1	0.3
			**Effective total**	**307**	**100.0**
			**Total (including missing values)**	**597**	
	Incorrect use of equipment		Lack of OHS training in workplaces	47	35.3
			Extemporaneous action	37	27.8
			Usual practice	35	26.3
			Communication	11	8.3
			Health status	3	2.3
			**Effective total**	**133**	**100.0**
			**Total (including missing values)**	**237**	
	Improper use of equipment		Usual practice	19	61.3
			Lack of OHS training in workplaces	6	19.4
			Extemporaneous action	5	16.1
			Communication	1	3.2
			**Effective total**	**31**	**100.0**
			**Total (including missing values)**	**71**	
		**Effective total**		**905**	
		**Total (including missing values)**		**919**	
Tools, Machines, Plants (N = 1900)	Set-up problems		Lack of equipment securities	792	49.5
			Insufficient equipment securities	221	13.8
			Presence of dangerous elements	81	5.1
			Removal of protections	64	4.0
			Tampering with protections	41	2.6
			Other set-up problems	401	25.1
			**Effective total**	**1600**	**100.0**
			**Total (including missing values)**	**1626**	
	Operating problems			274	
Materials (N = 346)	Storage			163	47.2
	Structural characteristics			148	42.9
	Transformations			34	9.9
	**Effective total**			**345**	**100.0**
	**Total (including missing values)**			**346**	
Environment (N = 1400)	Absence of barriers, protections, parapets, armor			516	36.9
	Inadequate signage			150	10.7
	Absence of safe routes			144	10.3
	Settlements, landslides, fall of heavy bodies			133	9.5
	Presence of bulky, dangerous elements			100	7.1
	Presence of electrical wires, electricity line			39	2,8
	Absence of suitable lighting			25	1.8
	Presence of gas, vapors			23	1.6
	Presence of liquids (water, oils, etc.)			20	1.4
	Other problems of the workplace			247	17.6
	**Effective total**			**1397**	**100.0**
	**Total (including missing values)**			**1400**	
PPE and clothing (N = 754)	Non-used yet available PPE			420	57.9
	PPE not provided			234	32.3
	Structural inadequacy			36	5.0
	Incorrect use			31	4.3
	Wear and tear			4	0.6
	**Effective total**			**725**	**100.0**
	**Total (including missing values)**			**754**	

**Table 7:** Two-way classification of fatal occupational accidents by number of factors originating the accidents (rows) and category of contributing factors (columns). Number (N), percentage (%). PPE, Personal protective equipment.

N. of Risk Factors		Categories of Contributing Factors							Accidents (Column %)
		Activity of Injured Person	Third–Party Activities	Tools, Machines, Plants	Materials	Environment	PPE and Clothing	All Categories	
**1**	**N**	938	140	252	51	206	19	1606	33.0
	(row %)	58.4	8.7	15.7	3.2	12.8	1.2	100.0	
**2**	N	2081	389	931	129	633	367	4530	46.5
	(row %)	45.9	8.6	20.6	2.8	14.0	8.1	100.0	
**3**	N	863	305	512	89	417	268	2454	16.8
	(row %)	35.2	12.4	20.9	3.6	17.0	10.9	100.0	
**4**	N	151	66	128	33	104	74	556	2.9
	(row %)	27.2	11.9	23.0	5.9	18.7	13.3	100.0	
**5**	N	33	18	47	23	38	26	185	0.8
	(row %)	17.8	9.7	25.4	12.4	20.5	14.1	100.0	
**6**	N	1	1	2	0	2	0	6	0.0
	(row %)	16.7	16.7	33.3	0.0	33.3	0.0	100.0	
**7**	N	0	0	28	21	0	0	49	0.1
	(row %)	0.0	0.0	57.1	42.9	0.0	0.0	100.0	
Total	N	4067	919	1900	346	1400	754	9386	100.0

**Table 8:** Frequencies of combination of two risk factors of occupational fatal injuries. Number (N), percentage (%). PPE, Personal protective equipment.

Risk Factors		Activity of the Injured Person	Activity of Third Parties	Tools, Machines, Plants	Materials	Environment	PPE and Clothing	Total *N* (Column %)
**Activity of the Injured Person**	*N*	405	572	1362	209	882	562	3.992
	(total %)	(6.7)	(9.4)	(22.4)	(3.4)	(14.5)	(9.2)	(65.6)
**Activity of Third Parties**	*N*		66	223	52	223	72	636
	(total %)		(1.1)	(3.7)	(0.9)	(3.7)	(1.2)	(10.5)
**Tools, Machines, Plants**	*N*			236	159	253	236	884
	(total %)			(3.9)	(2.6)	(4.2)	(3.9)	(14.5)
**Materials**	*N*				32	77	63	172
	(total %)				(0.5)	(1.3)	(1.0)	(2.8)
**Environment**	*N*					130	246	376
	(total %)					(2.1)	(4.0)	(6.2)
**PPE and Clothing**	*N*						25	25
	(total %)						(0.4)	(0.4)

Grey color: blank; Blue color: total number.

**Table 9:** Most frequent associations between two risk factors of fatal occupational accidents, by decreasing frequency. PPE, Personal protective equipment.

Risk Factors	Most Frequent Association for Fatal Events
Activity of the injured person	Tools, Machines, Plants
Activity of third parties	Activity of the injured person
Tools, Machines, Plants	Activity of the injured person
Materials	Activity of the injured person
Environment	Activity of the injured person
PPE and clothing	Activity of the injured person

## Data Availability

The datasets generated and analyzed during the current study are not publicly available but may be available from the corresponding author on reasonable request.
